# Relationship of Tubule Formation, Indian File Pattern and Apocrine Change With Estrogen and Progesterone Receptors and HER2 Immunostaining

**DOI:** 10.7759/cureus.30204

**Published:** 2022-10-11

**Authors:** Divya Singh, Akansha Agarwal, Michael L Anthony, Pranoy Paul, Monika Singh, Shalinee Rao, Bina Ravi, Nilotpal Chowdhury

**Affiliations:** 1 Pathology and Laboratory Medicine, All India Institute of Medical Sciences, Rishikesh, Rishikesh, IND; 2 Pathology and Laboratory Medicine, All India Institute of Medical Sciences, Gorakhpur, Gorakhpur, IND; 3 Pathology and Laboratory Medicine, India Institute of Medical Sciences, Rishikesh, Rishikesh, IND; 4 Surgery, SMSR (School of Medical Sciences and Research) Greater Noida, Greater Noida, IND; 5 Integrated Beast Care Center, All India Institute of Medical Sciences, Rishikesh, Rishikesh, IND

**Keywords:** erbb2, her2 positive breast carcinoma, receptors, estrogen, progesterone, breast carcinoma

## Abstract

Objectives: In breast carcinomas, histomorphological features like low-grade and lobular differentiation are associated with estrogen receptor (ER) and progesterone receptor (PR) expression. Apocrine carcinoma is associated with human epidermal growth factor receptor 2 (HER2) positivity. Studies have not emphasized the association between other histological features like tubule formation, Indian file pattern and apocrine change (which may be found in all grades of tumors or as a part of a mixed pattern of no special type) and immunohistochemistry (IHC). The study was designed to find the association between these morphological factors and ER, PR and HER2 status.

Materials and methods: The presence or absence of tubule formation, Indian file pattern and apocrine change was correlated with ER, PR and HER2 expression in core biopsies of 102 invasive breast carcinomas.

Statistical analysis: Fisher exact test with median unbiased odds ratio was used.

Results: Tubule formation and/or Indian file pattern were significantly associated with ER in all tumors (P-value <0.001), as well as separately for grade II, grade III, HER2-negative and HER2-positive tumors. Comparable results were obtained for their association with PR. Apocrine change was significantly associated with HER2 in all tumors (P-value <0.001), as well as separately for grade III, ER-positive and ER-negative tumors.

Conclusion: These histomorphological patterns are modest predictors of IHC status in breast carcinomas, even in tumors of higher grade. Knowledge of these morphological correlates of ER, PR and HER2 in breast cancer may serve as an aid in the quality management of breast carcinoma reporting.

## Introduction

Estrogen receptors (ERs), progesterone receptors (PRs) and human epidermal growth factor receptor 2 (HER2/neu or HER2) are established, essential predictive markers in breast cancer which help to choose systematic therapy. Knowledge of histomorphological types and features may aid in interpreting immunohistochemistry (IHC). They may be an important adjunct in identifying false-negative or false-positive immunostaining [1,2]. Studies have emphasized the positive association of ER/PR only with low-grade breast cancer, lobular carcinoma and low-grade special morphological types [3-6]. The negative association of HER2 with low grade and the positive association of HER2 with apocrine carcinomas have been emphasized [3,5,7]. Correlation of the microscopic features of breast cancer with IHC of ER, PR and HER2 is, therefore, recommended [1,2]. Such correlating histological features are limited, however, to only a small subset of breast cancers (low-grade carcinoma, some special types). Additional morphological features showing association with ER/PR or HER2 in breast cancers, especially in the higher grades, would provide further help in the correlation of the microscopic patterns of breast cancer with ER/PR and HER2.

Morphological features of tumors known to have a positive association with the IHC markers may be a good starting point to find out other correlating morphological features. Such correlating features may be, for example, the Indian file pattern, presence of tubules and presence of an apocrine component. The Indian file pattern is a feature of lobular differentiation which is known to be associated with ER/PR positivity [6]. Tubule formation is a feature of low-grade carcinoma, which is again associated with ER/PR positivity [5]. However, these features may be present as a minor component of grade II and grade III breast carcinoma of no special type. Apocrine change is a feature of apocrine carcinoma which is positively associated with HER2 status [8] but may coexist as a minor component of other types of breast carcinoma of any grade. We hypothesized that these morphological features would serve as markers of IHC status in breast cancers of any grade, including high-grade cancers. We did not find previous studies that have focused on the strength of the correlation of these morphological features (as opposed to histological type) with hormone receptor and HER2 expression. We set out to find whether these morphological features were associated with the IHC status of breast cancers, with special emphasis on high-grade (grade III) tumors and in IHC-defined subgroups.

## Materials and methods

This study was approved by the institutional ethics committee. One hundred and two biopsy sections, each from different patients with breast cancer, were examined for the presence of tubules, lobular differentiation and apocrine change. ER (Clone SP1, make Zytomed Systems, Berlin, Germany, Cat. No. BRB053, ready-to-use), PR (Clone SP42, make Zytomed Systems, Berlin, Germany, Cat. No. BRB038, ready-to-use) and HER2 (Clone QR003, make Quartett GmbH Berlin, Germany, ready-to-use) IHC were also examined and interpreted in all cases. The IHC staining was done as per the manufacturer’s instructions. It is the institutional protocol to immediately put the core in fixative. The time that the tissue is kept in the fixative ranges between 6 and 30 hours. Internal and external controls are examined during IHC assessment. ER and PR receptor positivity were defined as nuclear positivity in greater than 1% of tumor cells. HER2 was considered to be positive with a 3+ membranous staining. A 2+ staining was considered to be equivocal, while cases with 1+ or less staining were considered to be negative.

The positivity of hormone receptors (ER and PR) was tested against the presence of tubules, the presence of Indian files (indicating at least a “partial” targetoid pattern) and a combination of these two patterns. These patterns were evaluated irrespective of the predominant tumor type. The positivity of HER2 status was tested against the presence of cytoplasmic apocrine change. We considered the presence of a moderate amount of eosinophilic cytoplasm with prominent cytoplasmic granularity or vacuolization to correspond to cytoplasmic apocrine change. For the purpose of this study, this cytoplasmic apocrine change did not necessarily correspond to what is known as apocrine carcinoma with its characteristic nuclear changes and prominent nucleoli.

**Figure 1:**
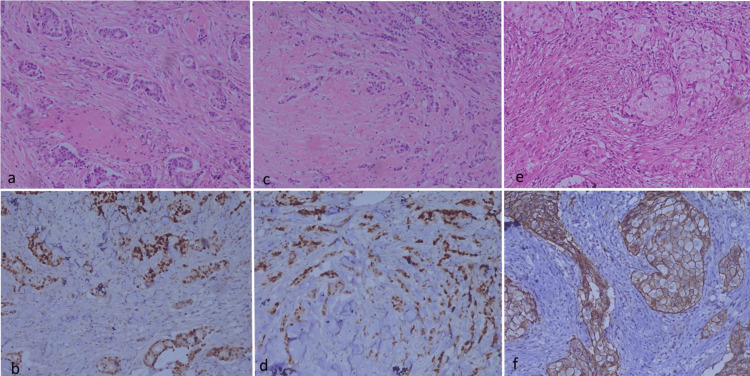
Showing tubule formation Showing tubule formation (a), ER staining in the same area (b), Indian file pattern (c), ER staining in the same area (d), and apocrine change (e), and HER2 staining of the same tumor (f). ER: estrogen receptor.

A representative illustration of the morphological patterns tested with IHC staining is shown in Figures 1a-1f. For each of these comparisons, the median unbiased odds ratio (OR) was estimated with the exact 95% confidence intervals. The statistical significance of the associations was tested with the exact mid-P-values. Sensitivity, specificity, predictive value for positive test (PPV) and predictive value for negative test (NPV) were also estimated for prediction of ER and PR by tubule formation, Indian file pattern and a combination of these two patterns, as well as for prediction of HER2 status by apocrine change. Statistical tests were performed using the R statistical environment, version 3.5.2 (R Foundation for Statistical Computing, Vienna, Austria), the R Commander plugin EZR (Jichi Medical University, Saitama Medical Center, Japan) and the “epitools” package (R package version 0.5-10.1, authored by Aragon T, available from https://CRAN.R-project.org/package=epitools).

## Results

The age of the 102 patients selected ranged from 20 years to 74 years with a median of 47 years. Two tumors were grade I, 27 were grade II and 69 were grade III, with four not graded due to scarcity of tissue. The size of the tumors ranged from 1.0 cm to 9.0 cm, with a median of 3.0 cm and a mean of 3.8 cm. Twenty-six tumors were ER positive/HER2 negative, 21 were ER positive/HER2 positive, 24 were ER negative/HER2 positive and 24 were triple negative. Seven tumors were HER2 equivocal, of which four were ER positive and three were ER negative. The HER2 equivocal tumors were classified as negative for all subsequent statistical analyses.

The odds ratio for all the examined morphological factors was markedly raised. The P-values were significant for the association between the tubule formation and presence of Indian files with hormone receptors (ER and PR) for all the tumors as well as for the subgroup of tumors showing apocrine change (Tables 1-3). A substantial majority (42/51) of the ER-positive tumors showed either a tubule formation or an Indian file pattern. A vast majority (42/45) of the HER2-positive tumors showed apocrine change.

**Table 1:** The relation between ER/PR with tubule formation and Indian file pattern along with the median unbiased odds ratio (OR) and the exact mid-P-value for association of all the breast cancers and the subgroups of tumors ER: estrogen receptor, PR: progesterone receptor, AC: apocrine change.

		Tubules and/or Indian files present		Tubules present		Indian files present	
	Comparison	OR (95% CI)	P-value	OR (95% CI)	P-value	OR (95% CI)	P-value
ER	All tumors	6.47 (2.67, 17)	<0.0001	2.84 (1.27, 6.55)	0.01	4.28 (1.89, 10.12)	0.0004
	Grade II	6.23 (1.07, 57.57)	0.04	2.03 (0.43, 10.45)	0.4	10.53 (1.89, 81.56)	0.006
	Grade III	5.05 (1.79, 15.72)	0.002	2.65 (0.97, 7.51)	0.06	3.06 (1.15, 8.56)	0.02
	HER2 -ve	4.79 (1.52, 16.86)	0.007	2.24 (0.77, 6.86)	0.1	3.30 (1.12, 10.32)	0.03
	HER2 +ve	9.16 (2.28, 50.15)	0.001	3.78 (1.11, 14.15)	0.03	5.72 (1.62, 22.91)	0.006
	AC absent	6.59 (0.89, 69.77)	0.06	1.41 (0.24, 8.39)	0.7	3.7 (0.63, 26.21)	0.2
	AC present	5.69 (2.09, 17.18)	0.0004	3.2 (1.25, 8.58)	0.02	4.21 (1.64, 11.5)	0.003
PR	All tumors	14.56 (4.58, 67.79)	<0.0001	4.36 (1.87, 10.64)	0.0006	5.45 (2.26, 14.25)	0.0001
	Grade II	4.96 (0.86, 45.21)	0.07	2.84 (0.59, 15.76)	0.2	7.4 (1.4, 51.73)	0.02
	Grade III	25.1 (4.59, 636.5)	<0.0001	3.4 (1.16, 10.42)	0.03	6.49 (2.01, 26.28)	0.001
	HER2 -ve	10.25 (2.43, 77.91)	0.0007	4.79 (1.52, 16.53)	0.007	3.22 (1.03, 11.15)	0.04
	HER2 +ve	20.69 (3.41, 550.67)	0.0002	3.70 (1.05, 14.36)	0.04	10.61 (2.60, 59.00)	0.0006
	AC absent	Infinity (2.01, Infinity)	0.003	7.74 (1.31, 61.92)	0.02	3.69 (0.65, 25.79)	0.1
	AC present	9.36 (2.77, 45.31)	0.0001	3.3 (1.23, 9.28)	0.02	5.75 (2.02, 18.55)	0.0008

**Table 2:** The relation between HER2 status and apocrine change, along with median unbiased odds ratio (OR) and the exact mid-P-value for association of all the breast cancers and the subgroups of tumors *Seven tumors were equivocal for HER2. ER: estrogen receptor.

	HER2 2+ tumors classified as HER2 negative*		HER2 2+ tumors removed from analysis	
	OR (95% CI)	P-value	OR (95% CI)	P-value
All tumors	8.30 (2.58, 38.90)	0.0001	7.44 (2.25, 35.35)	0.0005
Grade II	8.41 (1.14, 243.84)	0.04	7.39 (0.97, 216.63)	0.05
Grade III	10.61 (1.84, 273.44)	0.005	8.71 (1.41, 230.37)	0.02
ER -ve	7.01 (1.08, 189.99)	0.04	5.32 (0.73, 149.69)	0.1
ER +ve	8.64 (1.98, 66.85)	0.003	8.61 (1.92, 67.91)	0.003
Both tubules and Indian files absent	Infinity (1.16, Infinity)	0.02	Infinity (0.71, Infinity)	0.05
Tubules and/or Indian files present	6.01 (1.68, 30.09)	0.005	5.84 (1.59, 29.71)	0.007

**Table 3:** The sensitivity, specificity, predictive value for positive test (PPV) and predictive value for negative test (NPV) for the prediction of ER and PR by tubule and Indian file formation and for the prediction of HER2 status by apocrine change ER: estrogen receptor, PR: progesterone receptor.

	Sensitivity	Specificity	PPV	NPV
Tubule formation vs ER	0.569 (0.422, 0.707)	0.686 (0.541, 0.809)	0.644 (0.488, 0.781)	0.614 (0.476, 0.74)
Indian file pattern vs ER	0.686 (0.541, 0.809)	0.667 (0.521, 0.792)	0.673 (0.529, 0.797)	0.68 (0.533, 0.805)
Tubule and/or Indian file pattern vs ER	0.824 (0.691, 0.916)	0.588 (0.442, 0.724)	0.667 (0.537, 0.780)	0.769 (0.607, 0.889)
Tubule formation vs PR	0.658 (0.486, 0.804)	0.698 (0.57, 0.808)	0.568 (0.41, 0.717)	0.772 (0.642, 0.873)
Indian file pattern vs PR	0.763 (0.598, 0.886)	0.635 (0.504, 0.753)	0.558 (0.413, 0.695)	0.816 (0.68, 0.912)
Tubule and/or Indian file pattern vs PR	0.921 (0.786, 0.983)	0.571 (0.44, 0.695)	0.565 (0.433, 0.69)	0.923 (0.791, 0.984)
Apocrine change vs HER2	0.933 (0.817, 0.986)	0.386 (0.26, 0.524)	0.545 (0.428, 0.659)	0.88 (0.688, 0.975)

The Nottingham grade did not show a significant association with either ER or HER2 (P-value by Fisher’s exact test=0.4, 0.05 and 0.9 for the association with ER, PR and HER2, respectively). However, tubular and Indian file pattern continued to be predictive of ER and PR status, even in grade II and grade III tumors separately. Apocrine change was also significantly positively associated with HER2 status in grade III tumors, as well as in ER-positive tumors when HER2 2+ tumors were removed from the analysis; apocrine change was significantly positively associated with HER2 status in all analyzed subgroups when HER2 2+ tumors were classified as negative.

## Discussion

We found a strong correlation between morphological patterns and ER, PR and HER2 status. We found evidence of an association between the presence of tubule formation and/or Indian file pattern and ER/PR immune-staining even in high-grade tumors, where morphological patterns of differentiation are only focally present. Tubule formation and/or Indian file pattern were also found to be associated with ER positivity in tumors showing cytoplasmic apocrine change, despite apocrine differentiation being known to be negatively associated with hormone receptor positivity [8-10]. In both ER-positive tumors (likely to belong to luminal molecular subtype [11]) and ER-negative tumors (likely belonging to non-luminal molecular subtype), there was a positive association between cytoplasmic apocrine change and HER2 status. Identification of cytoplasmic apocrine change microscopically was predictive of HER2 status even in grade III tumors. Therefore, the association of morphological patterns with ER, PR and HER2 status is independent of the grade and type of the tumor. However, these patterns are sensitive, but not specific to the IHC status.

The correlation of ER and PR with tubule formation (in no special type (NST) breast cancers) and lobular carcinoma is long known [4], as is the association of apocrine carcinoma with HER2. Knowledge of these morphological correlates is interesting and useful in routine practice [1,2]. However, the present advice is to suspect the IHC results if ER or PR stains are negative in low-grade and lobular tumors or if HER2 stains are positive in a low-grade tumor. However, relying on only the (low) grade of a tumor to correlate the IHC status in order to suspect IHC staining quality issues addresses a small fraction of the tumors. This is because most breast carcinomas are morphologically heterogeneous grade II or III tumors. The present study demonstrates the usefulness of already known morphological concepts in the correlation of microscopic features with IHC even in high-grade tumors and in IHC-defined subgroups. These morphological patterns (tubules, Indian files and apocrine change) are a logical extension of our histopathological knowledge and should help in modestly predicting the IHC status even in the absence of a definite evidence of tumor type or in tumors having mixed morphological patterns.

The presence of a strong correlation between ER and PR with tubule formation and lobular differentiation as well as that between apocrine change and HER2 status may help in selecting the proper section for IHC, identifying batches of false-negative IHC and thus help in quality control [1]. There is some subjectivity associated with the recognition of Indian files as a “partial” targetoid pattern which possibly requires a pathologist's experience. Still, we believe that our results show it is worthwhile to recognize such a pattern. While lobular carcinoma is known to be associated with ER and PR expression [12], small areas may be admixed with a predominant NST tumor and missed, which may be even further exacerbated on the limited tissue in core biopsies.

We had a high number of tumors showing apocrine change and HER2 positivity, which may possibly be due to environmental factors associated with our particular geographical region. We intend to further investigate the cause of this increased proportion of apocrine differentiation and HER2 positivity, along with special molecular characteristics of the same. It was also our observation that this apocrine-like change was not only limited to the NST tumors but also to pleomorphic lobular carcinoma, a finding which is well known [9]. Interestingly, pleomorphic lobular carcinomas show a higher HER2 positivity than their classical counterparts, a finding which has been reported to be significantly associated with apocrine change [13]. Since HER2 positivity is inversely correlated with hormone receptor expression in breast carcinoma, this may have interfered with getting an even stronger correlation between the hormone receptor status and tubule formation or lobular differentiation.

Not testing in-situ hybridization for the HER2 equivocal tumors on IHC is a limitation of this study. We included only the IHC 3+ tumors as positive for HER2 amplification in our main analysis. However, similar odds ratios to the ones reported in the present study would be obtained even when HER2 equivocal tumors were removed from the analysis, with statistical significance at an alpha of 0.05. Therefore, we believe that the conclusions about the relationship between apocrine change and HER2 positivity are robust.

## Conclusions

We re-emphasize the role of histology as important, but imperfect, predictors of the IHC markers in breast cancers. This predictive ability is retained even if morphological typing is not possible. Attention to the histological features may serve as an important aid in maintaining the quality of IHC of routine breast markers.
